# Comprehensive Analysis of Differentially Expressed Profiles of mRNA, lncRNA, and circRNA in the Uterus of Seasonal Reproduction Sheep

**DOI:** 10.3390/genes11030301

**Published:** 2020-03-12

**Authors:** Yongfu La, Xiaoyun He, Liping Zhang, Ran Di, Xiangyu Wang, Shangquan Gan, Xiaosheng Zhang, Jinlong Zhang, Wenping Hu, Mingxing Chu

**Affiliations:** 1 Key Laboratory of Animal Genetics, Breeding and Reproduction of Ministry of Agriculture and Rural Affairs, Institute of Animal Science, Chinese Academy of Agricultural Sciences, Beijing 100193, China; layongfu@yeah.net (Y.L.); hedayun@sina.cn (X.H.); dirangirl@163.com (R.D.); xiangyu_wiggle@163.com (X.W.); 2 College of Animal Science and Technology, Gansu Agricultural University, Lanzhou 730070, China; zhanglp512@163.com; 3 State Key Laboratory of Sheep Genetic Improvement and Healthy Production, Xinjiang Academy of Agricultural and Reclamation Sciences, Shihezi 832000, China; shangquangan@163.com; 4 Tianjin Institute of Animal Sciences, Tianjin 300381, China; zhangxs0221@126.com (X.Z.); jlzhang1010@163.com (J.Z.)

**Keywords:** non-coding RNA, photoperiod, uterus, seasonal reproduction, sheep

## Abstract

Photoperiod is one of the important factors leading to seasonal reproduction of sheep. However, the molecular mechanisms underlying the photoperiod regulation of seasonal reproduction remain poorly understood. In this study, we compared the expression profiles of mRNAs, lncRNAs, and circRNAs in uterine tissues from Sunite sheep during three different photoperiods, namely, the short photoperiod (SP), short transfer to long photoperiod (SLP), and long photoperiod (LP). The results showed that 298, 403, and 378 differentially expressed (DE) mRNAs, 171, 491, and 499 DE lncRNAs, and 124, 270, and 400 DE circRNAs were identified between SP and LP, between SP and SLP, and between LP and SLP, respectively. Furthermore, functional enrichment analysis showed that the differentially expressed RNAs were mainly involved in the GnRH signaling pathway, thyroid hormone synthesis, and thyroid hormone signaling pathway. In addition, co-expression networks of lncRNA–mRNA were constructed based on the correlation analysis between the differentially expressed RNAs. Our study provides new insights into the expression changes of RNAs in different photoperiods, which might contribute to understanding the molecular mechanisms of seasonal reproduction in sheep.

## 1. Introduction

Reproduction has a critical impact on the profitability of sheep production, but seasonal reproduction is an important factor limiting the reproductive efficiency of sheep. Seasonal reproduction can be categorized as long-day (LD) breeders and short-day (SD) breeders [1], of which sheep are short-day breeders, and reducing the day length promotes the seasonal onset of the cycling activity [2]. Seasonal reproduction is strictly regulated by seasonal changes in relative day length and night length [3]. Recent studies have shown that, in mammals, light information is received by photoreceptors in the retina and transmitted to the pineal gland, where it inhibits the synthesis and secretion of melatonin, which is essential for seasonal reproduction [4,5,6,7]. In sheep, Small Tail Han sheep exhibit reproductive behavior all year round [8]. In contrast, Sunite sheep develop gonads and display seasonal reproductive behavior during specific times of the year [9]. Therefore, the molecular mechanism of seasonal reproduction of sheep can be better studied by using Sunite sheep as a model. The uterus plays an important role in the reproductive process of sheep and is also involved in the regulation of the estrous cycle, but the molecular mechanisms between the uterus and seasonal reproduction are not fully understood [10,11]. Therefore, an in-depth understanding of the molecular mechanisms of uterine related functions is important for studying the reproduction of sheep.

In recent years, long non-coding RNAs (lncRNAs) and circular RNAs (circRNAs) are considered to be key regulators because they play a crucial role in transcriptional regulation of gene expression and post-transcriptional levels [12,13]. LncRNAs are one class of ncRNAs that are more than 200 nucleotides in length and have no protein-coding potential [14,15]. Increasing evidence supports that lncRNA-mediated gene expression is critical in ram reproduction [16]. Circular RNAs (circRNAs) are a unique class of non-coding RNAs that are resistant to RNase degradation and have a stable structure due to the lack of 5′ and 3′ ends [17,18,19]. Although the function of animal circRNAs is still being indicated, some reports showed that circRNAs to sponge miRNAs regulate gene transcription, and regulate mRNAs stability [20,21]. There is increasing evidence that lncRNAs and circRNAs play important roles in the development of germ cells [22,23]. To date, the profiling of non-coding RNAs, especially circRNAs and their roles in the reproductive processes of the uterus, remain completely unknown.

In the present study, we performed transcriptome sequencing to examine mRNAs, lncRNAs, and circRNAs expression profiles in the uterus of sheep. We also conducted GO (Gene Ontology) and KEGG (Kyoto Encyclopedia of Genes and Genomes) pathway analyses and constructed co-expression networks. Our results demonstrate the molecular mechanisms that underlie the uterus’ regulation of reproduction, thus giving us newer insights regarding the regulation of seasonal reproduction in sheep.

## 2. Materials and Methods

### 2.1. Ethics Approval

All experiments were performed following the relevant guidelines and regulations set by the Ministry of Agriculture of the People’s Republic of China. Ethical approval on animal survival was given by the animal ethics committee of CAAS-IAS (No. IAS2018-3).

### 2.2. Experimental Animals and Sample Collection

Nine ewes were selected from Urat Middle Banner, Bayan Nur City, Inner-Mongolia Autonomous Region, China, and housed on a farm of the Tianjin Institute of Animal Sciences, Tianjin, China. These ewes were all approximately three years old and weighed 37 kg. All animals had free access to water and food. All animals were ovariectomized on 21 July under pentobarbital anesthesia and received a subcutaneous Silastic estradiol implant [1]. Estradiol treatment was achieved with an inner diameter of 3.35 mm and outer diameter of 4.65 mm, packed with 20 mg crystalline estradiol-17β (Sigma Chemical Co., St. Louis, MO, USA). The implant was inserted into the axillary region for 2 weeks and designed to produce circulated E levels approximately 3–5 pg/mL [24]. Finally, three Sunite sheep were moved into controlled photoperiod rooms for 42 days under an artificial short photoperiod (SP = 8 h light, 16 h dark); three Sunite sheep were moved into controlled photoperiod rooms for 42 days under an artificial long photoperiod (LP = 16 h light, 8 h dark); and three Sunite sheep were moved into long photoperiod controlled photoperiod rooms for 42 days after being moved into short photoperiod controlled photoperiod rooms for 42 days (SLP). Finally, all sheep were slaughtered, and the uterus tissue collected. All samples were immediately stored at −80 °C for total RNA extraction.

### 2.3. RNA Extraction, Library Construction, and RNA-seq

According to the manufacturer’s instruction, total RNA was extracted from the uteri using TRIzol (Invitrogen, Carlsbad, CA, USA). The RNA concentration, integrity, and quantity were assessed using a Kaiao K5500 spectrophotometer (Beijing Kaiao Technology Development Co., Ltd, Beijing, China) and a Bioanalyzer 2100 (Agilent, Santa Clara, CA, USA).

According to the manufacturer’s instruction, nine libraries (SP, *n* = 3; LP, *n* = 3; SLP, *n* = 3) were constructed from 3 μg of total RNA for per sample using NEB Next Ultra Directional RNA LibraryPrep Kit for Illumina (NEB, Ipswich, MA, USA). Before the generation of the libraries, the rRNAs were removed using Ribo-Zero™ Gold Kits (Epicentre, Madison, WI, USA). After cluster generation, the library preparations were sequenced on an Illumina Hiseq platform (Illumina, San Diego, CA, USA). Raw data of the performed RNA-seq have been recorded in the SRA public database (accession number: SRP241010).

### 2.4. Reference Genome Mapping and Transcriptome Assembly

Raw data in fastq format were processed through in-hoseperl scripts. In this step, clean reads were obtained by removing reads with adapter contamination, reads that contained poly-N, and low-quality reads from raw data. Simultaneously, the Q20, Q30, and GC contents of the clean data were calculated. All downstream analysis was based on high-quality clean data. HiSAT2 was used to align clean reads of each sample to the sheep reference genome *Oar v 4.0* [25]. StringTie was used for transcriptome assembly and reconstruction [26]. 

### 2.5. Identification of Potential lncRNA Candidates

LncRNAs were identified using the following workflow. (1) Transcripts > 200-nt long with >2 exons is obtained. (2) Transcripts with coverage less than 5 in all samples were removed. (3) The different classes of class_code annotated by “u”, “i”, and “x” were retained, which corresponded to lincRNAs, intronic lncRNAs, and anti-sense lncRNAs, respectively. (4) Used Gffcompare to compare with annotation files to screen out known mRNAs and other non-coding RNAs (e.g., rRNAs, tRNAs, snoRNAs, snRNAs). Transcripts without coding potential, as predicted by CNCI, CPC, PFAM, and CPAT, were candidate lncRNAs. 

### 2.6. Identification of circRNA

CIRI was an efficient and fast tool to identify circRNAs [27]. In order to ensure the reliability of other circRNAs, the BWA–MEM algorithm was used to perform the sequence splitting comparison, then the SAM file was scanned to find the PCC (paired chiastic clipping) and PEM (paired-end mapping) sites, as well as the GT-AG splicing signals [28]. Finally, the sequence with the junction site is re-aligned with the dynamic programming algorithm. CircRNAs were blasted against the circBase for annotation. Those that could not be annotated were defined as novel circRNAs. 

### 2.7. Analysis of Differentially Expressed (DE) Genes

The fragments per kilobase of transcript per million reads mapped (FPKM) value was used to estimate the expression levels of mRNAs and lncRNAs, while the spliced reads per billion mappings (SRPBM) value was utilized to determine the amount of circRNAs [29,30]. For experiments with three biological replicates, the differentially expressed lncRNAs, circRNAs, and mRNAs were identified using the R package DEseq2 after a negative binomial distribution [31]. We identified differentially expressed genes with a *p* < 0.01 and a fold change > 2.0 between two groups as differentially expressed lncRNAs and mRNAs, and a fold change > 2.0 and *p* < 0.05 between two groups as differentially expressed circRNAs.

### 2.8. Bioinformatics Analysis

The function of DE lncRNAs was predicted by the GO and KEGG analysis of their cis- and trans-target mRNAs, which were screened based on their genomic positional relation 50-kb upstream and downstream, for cis-target mRNAs, and based on the Pearson correlation coefficient of lncRNA-RNA pairs being ≥0.9, for trans-target mRNAs [32]. The function of DE circRNAs was revealed via GO and KEGG analysis of their parental genes. All genes were mapped to GO terms using the Gene Ontology database (http://www.geneontology.org), and then the functional enrichment analysis was performed using the KEGG biological pathways database (http://www.genome.jp). Enrichment analysis was performed on each term in GO and KEGG using a hypergeometric test. With the calculated *p* < 0.05 being defined as the significant threshold, the genes were screened and enriched for the pathways. Next, the significance of the term enrichment analysis was corrected by FDR, and the corrected *p*-value (Q-value) was obtained [33,34]. If a *p*-value was ≤0.05, enrichment was considered significant.

### 2.9. Co-Expression Network Construction

The co-expression network of common DE lncRNAs with their cis- and trans-target common DE mRNAs were constructed using the Cytoscape software (V3.1.1) to explore the function of key lncRNAs [35].

### 2.10. Gene Expression Validation by Quantitative Real-Time PCR

We used qRT-PCR to verify the gene expression levels. We used approximately 0.1 μg of each RNA sample and reverse transcribed it into cDNA using an RT reagent. Real-time PCR was performed at 95 °C for 10 min, followed by 95 °C for 15 s, 60 °C for 60 s for 45 cycles, and 72 °C for 30 s. qPCR was performed on the LightCycler 480 (Roche, Basel, Sweden) using the TB Green Real-time PCR Master Mix (TaKaRa, Dalian, China). β-Actin was used as an internal reference to normalize target gene expression. All primers used in the qRT-PCR are shown in Table S1. Each qPCR experiment was performed in triplicate, and the relative RNA expression values were calculated using the 2^−ΔΔCt^ method [36].

## 3. Results

### 3.1. Summary of Raw Sequence Reads

After removing low-quality sequences, a total of 348,470,686, 359,776,938, and 331,723,476 clean reads with greater than 93.91% of Q30 were obtained in SP, LP, and SLP, respectively (Table 1). Approximately 92% to 95% of the reads were successfully aligned to the *Ovis aries* reference genome (Table 1).

### 3.2. Differential Expression Analysis of mRNAs, lncRNAs, and circRNA

**Figure 1:**
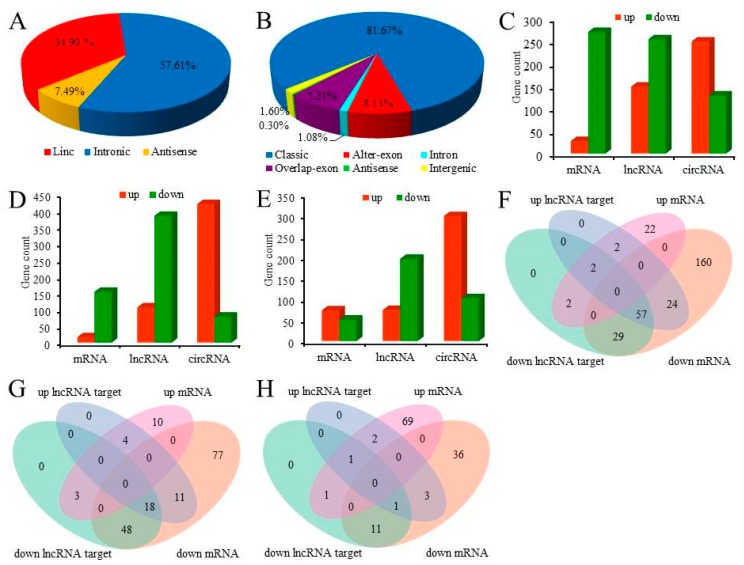
Gene expression characterization. (**A**) The type and proportion of lncRNAs. (**B**) The percentage of six types of circRNAs. (**C**) Histogram representing the numbers of upregulated and downregulated mRNAs and ncRNAs in sheep uteri between the short photoperiod (SP) and long photoperiod (LP) groups. (**D**) Histogram representing the numbers of upregulated and downregulated mRNAs and ncRNAs in sheep uteri between the SP and short transfer to long photoperiod (SLP) groups. (**E**) Histogram representing the numbers of upregulated and downregulated mRNAs and ncRNAs in sheep uteri between the LP and SLP groups. (**F**) Venn diagram representing the overlapping numbers of upregulated lncRNA-targeted mRNAs, downregulated lncRNA-targeted mRNAs, upregulated mRNAs, and downregulated mRNAs between the SP and LP groups. (**G**) Venn diagram representing the overlapping numbers of upregulated lncRNA-targeted mRNAs, downregulated lncRNA-targeted mRNAs, upregulated mRNAs, and downregulated mRNAs between the SP and SLP groups. (H) Venn diagram representing the overlapping numbers of upregulated lncRNA-targeted mRNAs, downregulated lncRNA-targeted mRNAs, upregulated mRNAs, and downregulated mRNAs between the LP and SLP groups.

A total of 19,996 mRNAs, 41,510 lncRNAs (including 2772 known lncRNAs and 38,738 novel lncRNAs), and 13,461circRNAs were identified from three groups (SP, LP, and SLP). The maximum proportion of intronic lncRNAs in the novel lncRNA was 57.61%, followed by lincRNAs for 34.90% and antisense lncRNAs for a minimum proportion of 7.49% (Figure 1A). There are six types of circRNA, of which classic circRNAs account for 81.67%, followed by overlap_exon circRNAs for 9.21%, and antisense circRNAs for a minimum proportion of 0.30% (Figure 1B). Three comparison groups were set according to the length of the illumination time, SP vs. LP, SP vs. SLP, and LP vs. SLP. For SP vs. LP, 28 mRNAs, 149 lncRNAs, and 249 circRNAs were upregulated, 270 mRNAs, 254 lncRNAs, and 129 circRNAs were downregulated (Figure 1C, Tables S2–S4). For SP vs. SLP, 17 mRNAs, 107 lncRNAs, and 420 circRNAs were upregulated, 154 mRNAs, 384 lncRNAs, and 79 circRNAs were downregulated (Figure 1D, Tables S5–S7). For LP vs. SLP, 73 mRNAs, 74 lncRNAs, and 298 circRNAs were upregulated, 51 mRNAs, 196 lncRNAs, and 102 circRNAs were downregulated (Figure 1E, Tables S8–S10). All the differentially expressed lncRNAs (*p* < 0.01), mRNAs (*p* < 0.01), and circRNA (*p* < 0.05) were statistically significant with a fold change greater than 2.0.

LncRNA regulated target gene (mRNAs) expression by Cis or Trans. If the target genes of the lncRNA are identical to the DE mRNAs, the DE mRNA may be further directly or indirectly regulated by lncRNAs. As shown in Figure 1F–H, the Venn diagram represents the intersectional analysis between the target mRNAs of the Cis or Trans with lncRNAs and DE mRNAs.

### 3.3. GO Analysis of the Biological Function of DE ncRNA

**Figure 2:**
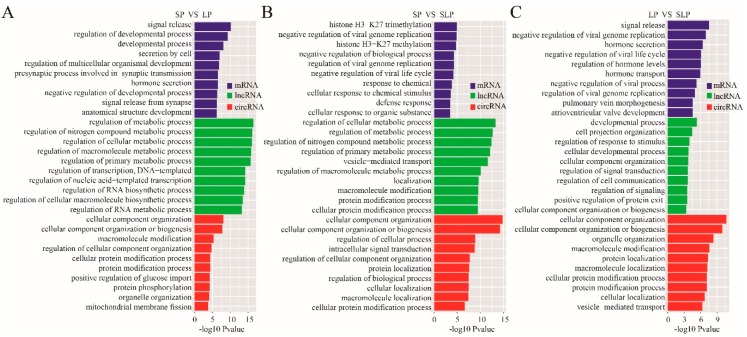
GO analyses of differentially expressed mRNAs, lncRNA targets, and circRNA host genes. (**A**) The top 10 enrichment biological processes for differentially expressed mRNAs, lncRNA targets, and circRNA host genes are listed between the SP and LP groups. (**B**) The top 10 enrichment biological processes for differentially expressed mRNAs, lncRNA targets, and circRNA host genes are listed between the SP and SLP groups. (**C**) The top 10 enrichment biological processes for differentially expressed mRNAs, lncRNAs target, and circRNA host genes are listed between the LP and SLP groups.

GO annotation enrichment was used to describe functions of the DE ncRNA involved in cellular components, molecular function, and biological processes. As shown in Figure 2, the GO enrichment analysis shows the top 10 GO terms. Between SP and LP, the DE mRNAs were most enriched, and the meaningful terms were related to the regulation of the developmental process, developmental process, and secretion by the cell. The targeted genes for DE lncRNAs were most enriched, and the terms were related to the regulation of cellular metabolic process and regulation of the metabolic process. The sourced genes for DE circRNAs were the most enriched terms and were related to cellular component organization and macromolecule modification (Figure 2A, Tables S11–S13).

Between SP and SLP, the DE mRNAs were most enriched, and the meaningful terms were related to negative regulation of biological process and cellular response to an organic substance. The targeted genes for DE lncRNAs were most enriched, and the terms were related to the regulation of the cellular metabolic process, regulation of the metabolic process, and regulation of the primary metabolic process. The sourced genes for DE circRNAs were most enriched, and the terms were related to cellular component organization and regulation of cellular process (Figure 2B, Tables S14–S16).

Between LP and SLP, the DE mRNAs were most enriched, and the meaningful terms were related to the regulation of hormone levels, regulation of secretion by the cell, and hormone secretion. The targeted genes for DE lncRNAs were most enriched, and terms were related to the developmental process and cellular developmental process. The sourced genes for DE circRNAs were most enriched, and the terms were related to cellular component organization and organelle organization (Figure 2C, Tables S17–S19).

### 3.4. KEGG Pathway Analysis

**Figure 3:**
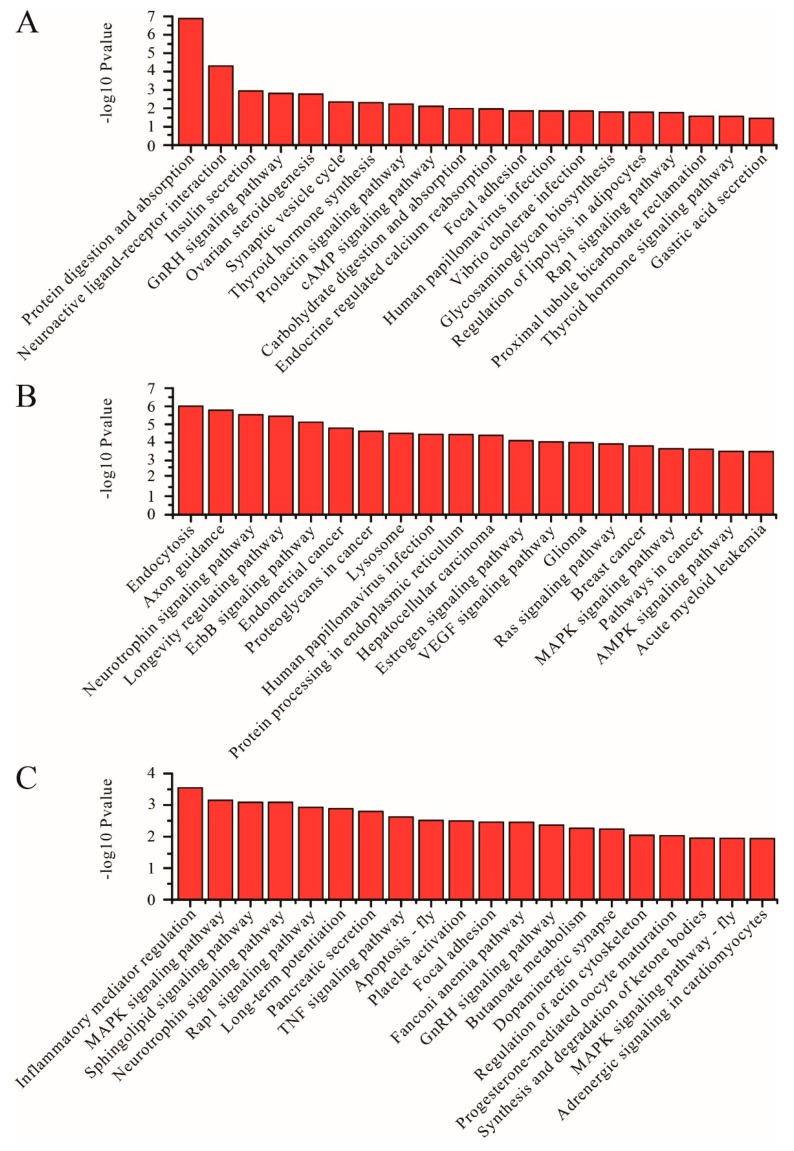
KEGG analyses of differentially expressed genes between the SP and LP groups. (**A**) The top 20 KEGG enrichment pathways for differentially expressed mRNAs between the SP and LP groups. (**B**) The top 20 KEGG enrichment pathways for differentially expressed lncRNA target genes between the SP and LP groups. (**C**) The top 20 KEGG enrichment pathways for differentially expressed circRNA host genes between the SP and LP groups.

**Figure 4:**
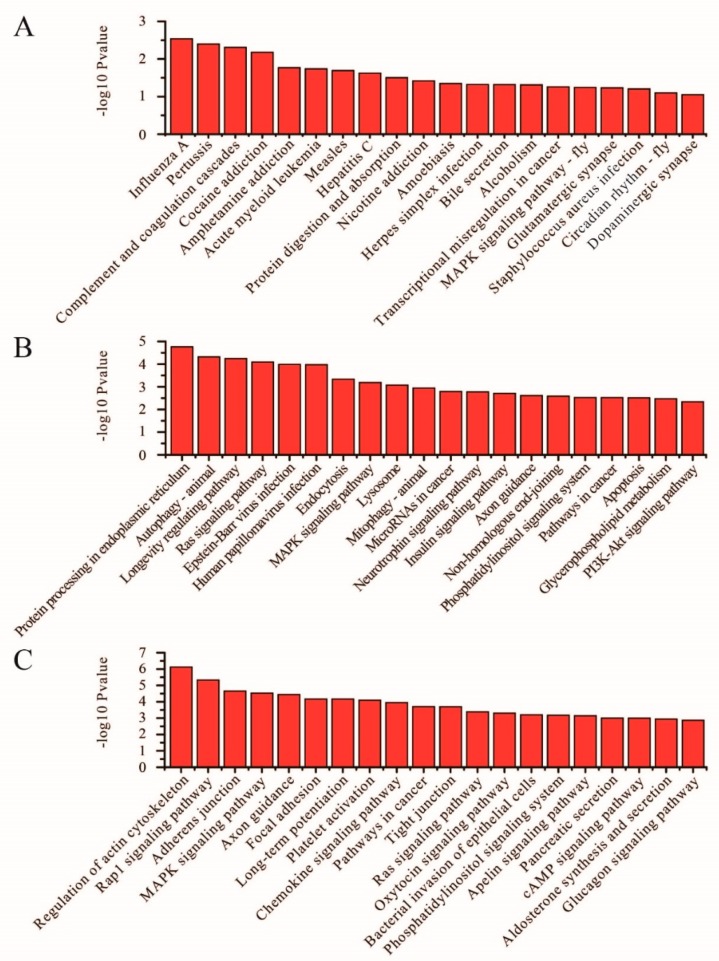
KEGG analyses of differentially expressed genes between the SP and SLP groups. (**A**) The top 20 KEGG enrichment pathways for differentially expressed mRNAs between the SP and SLP groups. (**B**) The top 20 KEGG enrichment pathways for differentially expressed lncRNA target genes between the SP and SLP groups. (**C**) The top 20 KEGG enrichment pathways for differentially expressed circRNA host genes between the SP and SLP groups.

**Figure 5:**
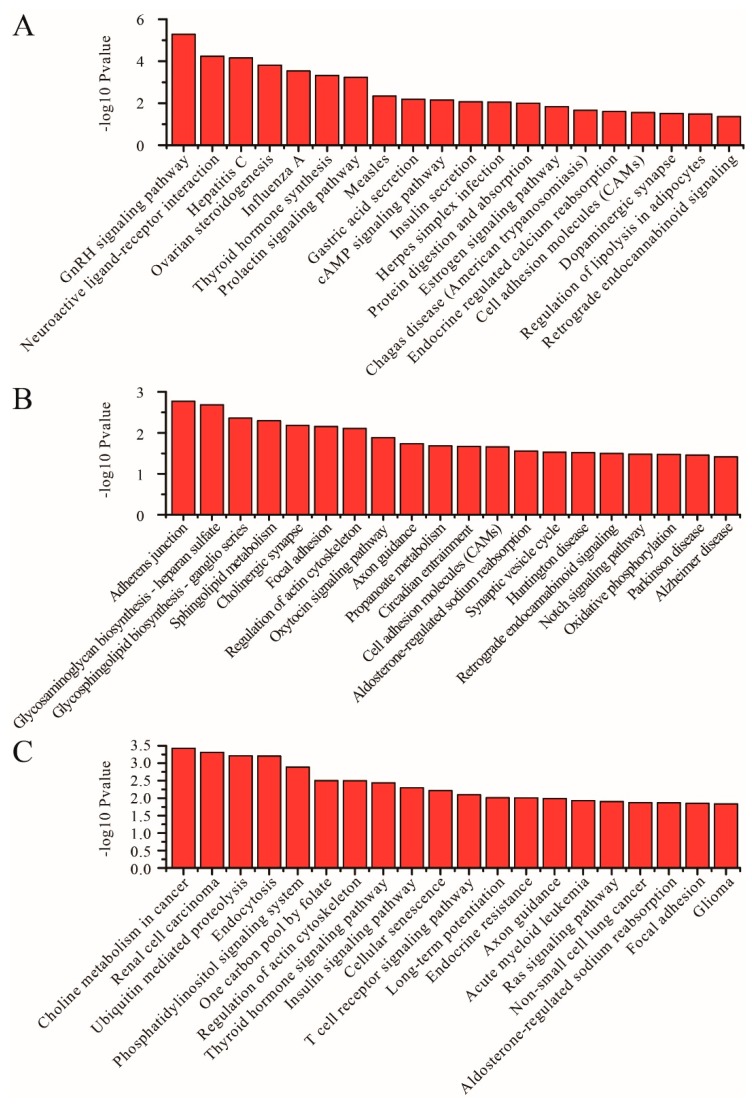
KEGG analyses of differentially expressed genes between the LP and SLP groups. (**A**) The top 20 KEGG enrichment pathways for differentially expressed mRNAs between the LP and SLP groups. (**B**) The top 20 KEGG enrichment pathways for differentially expressed lncRNA target genes between the LP and SLP groups. (**C**) The top 20 KEGG enrichment pathways for differentially expressed circRNA host genes between the LP and SLP groups.

KEGG is a primary public pathway database. The graphic exhibition of KEGG enrichment analysis represents the augmented scatter diagram of the selected target genes. The top 20 pathways are shown in Figure 3, Figure 4 and Figure 5. Between SP and LP, the DE mRNAs were enriched in protein digestion and absorption, insulin secretion, GnRH signaling pathway, ovarian steroidogenesis, thyroid hormone synthesis, prolactin signaling pathway, cAMP signaling pathway, carbohydrate digestion and absorption, and thyroid hormone signaling pathway (Figure 3A, Table S20). With regard to differentially expressed lncRNAs, targeted mRNAs were associated with pathways such as the estrogen signaling pathway and the VEGF signaling pathway (Figure 3B, Table S21). With regard to differentially expressed circRNA, host genes were associated with pathways such as GnRH signaling pathway and starch and sucrose metabolism (Figure 3C, Table S22).

Between SP and SLP, the DE mRNAs were enriched in protein digestion and absorption, alanine aspartate glutamate metabolism, and aldosterone synthesis and secretion (Figure 4A, Table S23). With regard to differentially expressed lncRNA, targeted mRNAs were associated with pathways such as protein processing in the endoplasmic reticulum and the MAPK signaling pathway (Figure 4B, Table S24). With regard to differentially expressed circRNA, host genes were associated with pathways such as the MAPK signaling pathway and fat digestion and absorption (Figure 4C, Table S25).

Between LP and SLP, the DE mRNAs were enriched in the GnRH signaling pathway, ovarian steroidogenesis, thyroid hormone synthesis, prolactin signaling pathway, cAMP signaling pathway, insulin secretion, protein digestion and absorption, and estrogen signaling pathway (Figure 5A, Table S26). With regard to differentially expressed lncRNAs, targeted mRNAs were associated with pathways such as sphingolipid metabolism, oxytocin signaling pathway, and propanoate metabolism (Figure 5B, Table S27). With regard to differentially expressed circRNAs, host genes were associated with pathways such as ubiquitin mediated proteolysis and fat digestion and absorption (Figure 5C, Table S28).

### 3.5. Co-Expression of lncRNAs-mRNAs and Function Prediction

**Figure 6:**
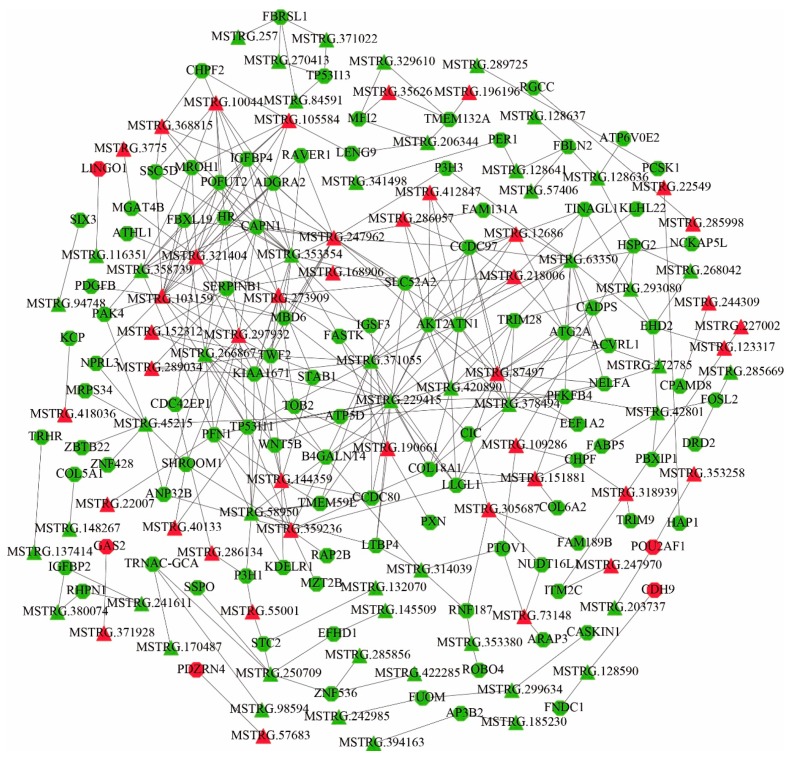
Construction of the lncRNA–mRNA co-expression network between the SP and LP groups. Red and green represent upregulated and downregulated, respectively. Octagons and triangles represent mRNAs and lncRNAs, respectively.

To explore the molecular mechanism of the effect of illumination time on sheep estrus, a co-expression network was constructed based on the expression levels of DE lncRNAs and DE mRNAs. In the SP and LP groups, a total of 117 DE mRNAs and 93 DE lncRNAs were involved in the network, and it consisted of 383 edges (Figure 6, Table S29). The top 5 upregulated expressed DE mRNAs are *LOC101105553*, *CDH9*, *PDZRN4*, *POU2AF1*, and *GAS2*, and the top 5 downregulated expressed DE mRNAs are *SIX3*, *TRHR*, *AP3B2*, *CADPS*, and *PCSK1*. These genes involved many functions, such as regulation of the developmental process, development, pituitary gland development, secretion, and protein metabolic process. 

**Figure 7:**
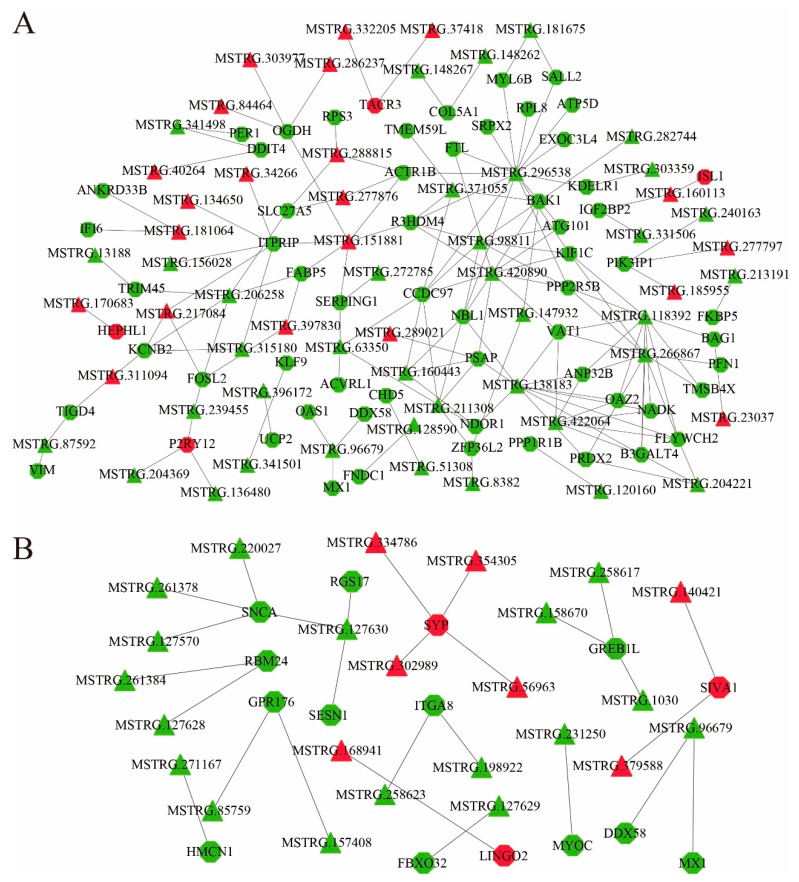
Construction of the lncRNA–mRNA co-expression network. (**A**) Construction of the lncRNA–mRNA co-expression network between the SP and SLP groups. (**B**) Construction of the lncRNA–mRNA co-expression network between the LP and SLP groups. Red and green represent upregulated and downregulated, respectively. Octagons and triangles represent mRNAs and lncRNAs, respectively.

In the SP and SLP groups, a total of 84 DE mRNAs and 69 DE lncRNAs were involved in the network, and it consisted of 216 edges (Figure 7A, Table S30). The top 5 upregulated expressed DE mRNAs are *ISL1*, *LIN7A*, *HEPHL1*, *LOC101108321*, and *P2RY12*, and the top 5 downregulated expressed DE mRNAs are *ABCG8*, *LOC101121593*, *EXOC3L4*, *LOC101102110*, and *IFI6*. These genes involved many functions, such as Signaling pathways regulating pluripotency of stem cells, developmental process, ABC transporters, Cholesterol metabolism, and Cellular senescence. 

In LP and SLP groups, a total of 19 DE mRNAs and 29 DE lncRNAs were involved in the network, and it consisted of 32 edges (Figure 7B, Table S31). The top 5 upregulated expressed DE mRNAs are *LOC101114852*, *SYP*, *SIVA1*, and *LINGO2*, and the top 5 downregulated expressed DE mRNAs are *RGS17*, *MX1*, *SNCA*, *LOC101105260*, and *MYOC*. These genes involved many functions, such as regulation of the developmental process, developmental process, regulation of secretion, and regulation of signaling.

### 3.6. Validation of Sequencing Data by qRT-PCR

**Figure 8:**
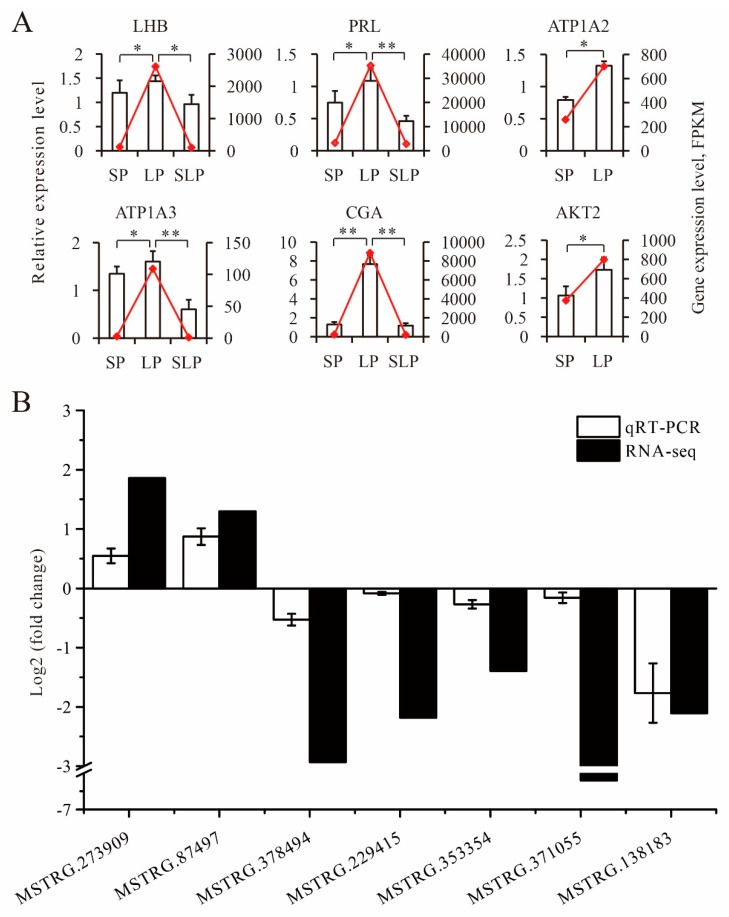
Validation of the expression of differentially expressed genes by qRT-PCR. (**A**) Validation of the expression of differentially expressed mRNAs by qRT-PCR. Data from the qRT-PCR are shown as columns and on the Y-axis on the left, while the data from RNA-Seq are shown as lines and on the Y-axis on the right. Data are represented as the mean ± SD, *n* = 3 per group. * *p* < 0.05, ** *p* < 0.01. (**B**) Validation of the expression of differentially expressed lncRNAs by qRT-PCR.

A total of thirteen genes, including six mRNAs (*LHB*, *PRL*, *ATP1A2*, *ATP1A3*, *CGA*, and *AKT2*) related to reproduction and seven random lncRNAs (MSTRG.273909, MSTRG.87497, MSTRG.378494, MSTRG.229415, MSTRG.353354, MSTRG.371055, and MSTRG.138183), were selected for qRT-PCR verification. The qRT-PCR analysis showed that the expression trends in the genes were similar to the trends in the RNA-seq results, supporting the credibility of the transcriptomics data (Figure 8A,B).

## 4. Discussion

Seasonal reproduction is the result of the adaptation of animal reproductive activities to environmental changes that are essential for breeding success and survival of future generations [37]. Thus, its molecular mechanism is worth researching. More and more evidence has shown that ncRNAs play critical roles in reproductive mechanisms [38,39,40]. However, comprehensive analyses of the profiles of differentially expressed lncRNAs and circRNAs in the uterus of the seasonal reproduction sheep have not yet been studied. So, we explored the expression profiles and predicted the potential functions of lncRNAs and circRNAs in the uterus of the seasonal reproduction sheep using RNA-Seq and bioinformatics analysis.

GO and KEGG pathway enrichment analyses showed that differentially expressed RNAs were associated with pathways such as the GnRH signaling pathway, thyroid hormone synthesis, cAMP signaling pathway, ovarian steroidogenesis, prolactin signaling pathway, carbohydrate digestion and absorption, and thyroid hormone signaling pathway. There has been increasing evidence that seasonal reproduction is regulated through the hypothalamic-pituitary-thyroid (HPT) axis and the hypothalamic-pituitary-gonadal (HPG) axis [3,41,42,43]. In the HPT axis, thyrotropin-releasing hormone (TRH) secreted from the hypothalamus induces the pituitary to release thyroid-stimulating hormone (TSH), which in turn stimulates the thyroid gland to synthesize and release TH [3,43]. In the HPG axis, GnRH is secreted from the hypothalamus and stimulates the release of luteinizing hormone and follicle-stimulating hormone. These hormones act on the gonads, promoting gonadal development and the production of steroid hormones [42,44,45,46]. Seasonal breeders activate the HPG axis through TH during the breeding season to regulate gonad development [47,48]. Therefore, the GnRH signaling pathway, thyroid hormone signaling pathway, cAMP signaling pathway, ovarian steroidogenesis, prolactin signaling pathway, and their related genes, are very important for seasonal reproduction.

In this study, differentially expressed genes *CGA*, *LOC101102411*, *ATP1A3*, *SLC26A4*, *ATP1A2*, *AKT2*, and *NOTCH4* were enriched in the thyroid hormone signaling pathway. Meanwhile, *ATP1A2*, *ATP1A3*, *LOC101102411*, *AKT2*, *DRD2*, *FSHB*, *ARAP3*, *MAPK10*, and *VIPR2* were enriched in the cAMP signaling pathway. Compared with the SP groups, *CGA*, *LOC101102411*, *AKT2*, *DRD2*, *FSHB*, *ARAP3*, *NOTCH4*, *ATP1A2*, and *ATP1A3* were upregulated in uterine tissue in the LP groups. Compared with the LP groups, *CGA*, *LOC101102411*, *ATP1A3*, *ATP1A2*, *FSHB*, *AKT2*, and *MAPK10* were downregulated in uterine tissue in the SLP groups. It has been reported that *CGA* expression has a robust photoperiodic response in melatonin-proficient CBA/N mice [49]. In quail, the expression of *CGA* was upregulated under long-term exposure to long-day conditions [50]. Similarly, in this study, *CGA* was significantly upregulated in LP compared with SP, and significantly downregulated in SLP compared with LP, indicating that *CGA* expression was proportional to photoperiod. *ATP1A3* is predominantly expressed in photoreceptor cells and optic nerve fibers, and *ATP1A2* is mainly expressed in retinal glial cells and astrocytes in the optic nerve [51]. In mammals, light information is received by photoreceptors in the retina and neurally transmitted to the pineal gland, where it inhibits the synthesis and secretion of melatonin, which is crucial for seasonal reproduction [3]. *ATP1A2* and *ATP1A3* are significantly upregulated in LP, suggesting that *ATP1A2* and *ATP1A3* may regulate the light information in photoreceptors by positive feedback, thereby inhibiting the synthesis and secretion of melatonin. Melatonin is a pleiotropic molecule that plays an important role in the seasonal reproduction of animals [52]. There has been a study showing that the presence of melatonin during oocyte maturation under the heat stress increased the gene expressions of *AKT2* [53]. Similarly, in this study, *AKT2* was significantly upregulated in LP compared with SP, and significantly downregulated in SLP compared with LP, indicating that *AKT2* expression was proportional to the photoperiod. The above studies show that *AKT2* plays an important role in the seasonal reproduction of sheep.

More importantly, compared with SP, we discovered that the common DE mRNAs during the entire photoperiod process were mainly involved in the GnRH signaling pathway, ovarian steroidogenesis, prolactin signaling pathway, and cAMP signaling pathway. The levels of many well-known key markers, such as *GnRHR*, *LHB*, and *FSHB*, for the GnRH signaling pathway and neuroactive ligand-receptor interaction, and *LHB* and *FSHB* for ovarian steroidogenesis and ovarian steroidogenesis, and *LHB* for the prolactin signaling pathway, and *FSHB* for the cAMP signaling pathway, significantly changed during the entire photoperiod process, suggesting that these pathways might play critical roles in seasonal reproduction.

LncRNAs and circRNAs are drawing increased attention as the most popular ncRNAs, and they participate in the regulation of different biological processes in different ways [54,55,56]. In this study, it was found that lncRNA and circRNA alterations are involved in the regulatory mechanisms of seasonal reproduction. A large number of differentially expressed lncRNAs and circRNAs were identified. GO and KEGG pathway analyses predicted that these differentially expressed lncRNAs and circRNAs are functionally related to hormonal regulation and metabolism-related pathways. More importantly, significantly differentially expressed lncRNAs targeted significantly differentially expressed mRNAs and were associated with the developmental process, pituitary gland development, regulation of secretion, and protein metabolic process. For example, lncRNA MSTRG.94748 was predicted to act on *SIX3* through cis-targeting. *SIX3* is expressed in an immature gonadotrope cell line and inhibits transcription of common α-subunit (*Cga*) and *GnRHR* genes during an early developmental stage [57]. In turn, lncRNA MSTRG.229415, MSTRG.247962, MSTRG.286057, MSTRG.371055, MSTRG.378494, MSTRG.420890, MSTRG.63350, and MSTRG.87497 were predicted to act on *AKT2* through trans-targeting. *AKT2* is a serine/threonine kinase and is necessary for a blastocyst’s basic glucose metabolism; it is essential for implantation in the maternal endometrium [58,59]. The lncRNA MSTRG.137414 was predicted to act on *TRHR* through trans-targeting. *TRHR* is an important element regulating THs synthesis and release, while seasonal breeders activate the HPG axis through TH during the breeding season to regulate gonadal development [48,60]. Mitogen-activated Protein Kinase Kinase Kinase 2 (*MAP3K2*) is an upstream MAPK kinase of the MAPK signaling pathway that is targeted by oar_circ_0001714 and plays a critical role in cell proliferation, differentiation, and cell migration [61,62]. From these data, it is inferred that the identified DE lncRNAs and DE circRNAs play a critical role in the seasonal reproduction of sheep.

## 5. Conclusions

In summary, our study provided a genome-wide view of the expression profiling of mRNAs, lncRNAs, and circRNAs in sheep uteri during different photoperiods. Moreover, a large number of DE genes that may affect seasonal reproduction in sheep under different photoperiods were further identified. We also predicted the potential role of these differentially expressed ncRNAs and constructed the mRNA–lncRNA network to expand our understanding. Our study provides a comprehensive basis of the expression levels of various RNAs in different photoperiods, providing new clues for understanding the mechanism of the molecular regulation of seasonal reproduction in sheep.

## Figures and Tables

**Table 1:** Summary of raw reads after quality control and mapping to the reference genome.

Sample	Raw Reads Number	Clean Reads Number	Clean Reads Rate (%)	Q30 (%)	Mapped Reads	Mapping Rate (%)
SP1	104,864,024	101,875,618	97.15	93.91	96,768,407	94.99
SP2	130,427,488	125,292,334	96.06	94.32	119,025,677	95.00
SP3	125,150,904	121,302,734	96.93	94.32	115,367,048	95.11
LP1	126,830,006	121,409,220	95.73	94.32	115,305,056	94.97
LP2	124,118,790	116,375,640	93.76	94.45	108,202,328	92.98
LP3	126,582,542	121,992,078	96.37	94.07	115,470,412	94.65
SLP1	111,749,300	106,892,084	95.65	94.15	100,913,471	94.41
SLP2	110,648,428	107,487,864	97.14	94.17	101,887,502	94.79
SLP3	121,906,706	117,343,528	96.26	94.35	110,324,951	94.02

## Supplementary Materials

Supplementary materials can be found at https://www.mdpi.com/2073-4425/11/3/301/s1. Table S1: Details of primer sequences and expected product sizes of genes used for qRT-PCR, Table S2: Differentially expressed mRNAs between SP and LP, Table S3: Differentially expressed lncRNAs between SP and LP, Table S4: Differentially expressed circRNAs between SP and LP, Table S5: Differentially expressed mRNAs between SP and SLP, Table S6: Differentially expressed lncRNAs between SP and SLP, Table S7: Differentially expressed circRNAs between SP and SLP, Table S8: Differentially expressed mRNAs between LP and SLP, Table S9: Differentially expressed lncRNAs between LP and SLP, Table S10: Differentially expressed circRNAs between LP and SLP, Table S11: The differentially expressed mRNAs GO enrichment analyses between SP and LP, Table S12: The differentially expressed lncRNAs targeted genes GO enrichment analyses between SP and LP, Table S13: The differentially expressed circRNAs sourced genes GO enrichment analyses between SP and LP, Table S14: The differentially expressed mRNAs GO enrichment analyses between SP and SLP, Table S15: The differentially expressed lncRNAs targeted genes GO enrichment analyses between SP and SLP, Table S16: The differentially expressed circRNAs sourced genes GO enrichment analyses between SP and SLP, Table S17: The differentially expressed mRNAs GO enrichment analyses between LP and SLP, Table S18: The differentially expressed lncRNAs targeted genes GO enrichment analyses between LP and SLP, Table S19: The differentially expressed circRNAs sourced genes GO enrichment analyses between LP and SLP, Table S20: KEGG enrichment analyses of mRNAs between SP and LP, Table S21: KEGG enrichment analysis of the targeted genes of differentially expressed lncRNAs between SP and LP, Table S22: KEGG enrichment analysis of the sourced genes of circRNAs differentially expressed between SP and LP, Table S23: KEGG enrichment analyses of mRNAs between SP and SLP, Table S24: KEGG enrichment analysis of the targeted genes of differentially expressed lncRNAs between SP and SLP, Table S25: KEGG enrichment analysis of the sourced genes of circRNAs differentially expressed between SP and SLP, Table S26: KEGG enrichment analyses of mRNAs between LP and SLP, Table S27: KEGG enrichment analysis of the targeted genes of differentially expressed lncRNAs between LP and SLP, Table S28: KEGG enrichment analysis of the sourced genes of circRNAs differentially expressed between LP and SLP, Table S29: The network that differentially expressed lncRNAs and their targeted differentially expressed mRNAs between SP and LP, Table S30: The network that differentially expressed lncRNAs and their targeted differentially expressed mRNAs between SP and SLP, Table S31: The network that differentially expressed lncRNAs and their targeted differentially expressed mRNAs between LP and SLP.

## Abbreviations

SP	short photoperiod
LP	long photoperiod
SLP	short transfer to long photoperiod
DE	differentially expressed
SD	short-day
LD	long-day
LncRNAs	long non-coding RNAs
CircRNAs	circular RNAs
qRT-PCR	quantitative real-time polymerase chain reaction
PCC	paired chiastic clipping
PEM	paired-end mapping
FPKM	fragments per kilobase of transcript per million read mapped
SRPBM	spliced reads per billion mappings
FDR	false discovery rate
